# His-Purkinje system pacing reduced tricuspid regurgitation in patients with persistent atrial fibrillation after left-sided valve surgery

**DOI:** 10.3389/fcvm.2023.1049482

**Published:** 2023-03-07

**Authors:** Ning Wang, Tianyu Zhu, Yan Li, Guanliang Cheng, Yu Chen, Yuwei Fu, Xuezhi Chen, Xiaohui Liu

**Affiliations:** ^1^ Department of Cardiology, Peking University International Hospital, Beijing, China; ^2^ Department of Ultrasound, Peking University International Hospital, Beijing, China

**Keywords:** his bundle pacing, left bundle branch pacing, tricuspid regurgitation, atrial fibrillation, heart valve disease.

## Abstract

**Background & objective:**

Tricuspid regurgitation after left-sided valve surgery was a common and difficult problem. Atrial fibrillation was considered to be an important etiology of tricuspid regurgitation. His-Purkinje system pacing (HPSP) was a physiological pacing method, which could prevent and treat heart failure and might reduce tricuspid regurgitation. Our study aimed to investigate the effect of HPSP on tricuspid regurgitation in patients with persistent atrial fibrillation after left-sided valve surgery.

**Methods:**

This study was a retrospective study. The 3-year patient review focused on those who underwent permanent cardiac pacemaker implantation of HPSP after mitral valve and/or aortic valve replacement from Jan 1st, 2019 to Jan 1st, 2022. HPSP included His bundle pacing (HBP) or left bundle branch pacing (LBBP). Clinical data collected included electrocardiogram, pacing parameters, ultrasonic cardiogram parameters and chest x-ray at implantation and 3-month follow up. Univariate and multivariate linear regression analysis of tricuspid regurgitation velocity were performed.

**Results:**

A total of 44 patients was retrospectively reviewed. Eight patients who had undergone implantation of HPSP after left-sided heart valve replacement were enrolled in the study. All patients had persistent atrial fibrillation. Three of them received HBP and five underwent LBBP. At 3-month follow-up, the tricuspid regurgitation grade was significantly lower than that before implantation (*P *= 0.007). The tricuspid regurgitation velocity significantly decreased (317 ± 74 cm/s vs. 261 ± 52 cm/s, *P = 0.022*) and tricuspid valve pressure gradient (PG) reduced (42 ± 21 mmHg vs. 28 ± 10 mmHg, *P = 0.040*). The cardiothoracic ratio of patients was significantly lower than that before implantation (0.61 ± 0.08 vs. 0.64 ± 0.09, *P = 0.017*). The NYHA classification of patients also improved (*P *= 0.013). In multivariate liner regression analysis, the pacing ratio (*β *= 0.736, *P = 0.037*) was an independent determinant of tricuspid regurgitation velocity variation.

**Conclusion:**

HPSP might reduce tricuspid regurgitation and improve cardiac function in patients with persistent atrial fibrillation after left-sided valve surgery.

## Introduction

Left-sided valve surgery was a common procedure for left heart valve (mitral valve, and aortic valve) disease. About 69,000 patients in China underwent valve replacement or repair operations every year (1). Secondary tricuspid regurgitation was a common comorbidity in patients who had left-sided valve replacement. Its incidence increased over time to more than 50% (2). The progressive aggravating tricuspid regurgitation gradually deteriorates the heart function and eventually leads to refractory right heart failure (3). Although these patients could take another tricuspid valve surgery, the timing for surgery was difficult to determine, and many patients were delayed until the end-stage, losing the opportunities for surgery (4). Furthermore, the mortality of tricuspid valve surgery after the first left-sided valve surgery could reach 25%, and the three-year survival rate was only 19% (5). Therefore, finding treatment strategies for preventing the progression of tricuspid regurgitation was the best hope for reducing the mortality and morbidity for these patients.

More than 90% of the patients after left-sided valve surgery had persistent atrial fibrillation (AF) (3), which could promote the dilatation of the tricuspid annulus, considered to be an important etiology of exacerbation of tricuspid regurgitation (6). When atrial fibrillation was combined with bradycardia, permanent pacemaker implantation was required (7). The His-Purkinje system pacing (HPSP) including His bundle pacing (HBP) and left bundle branch pacing (LBBP) was a new physiological pacing method, which can prevent and treat heart failure by directly pacing the His bundle or the left bundle branch to maintain synchronous pacing (8, 9). A recently published systematic review showed that there was marked improvement in tricuspid regurgitation grade after His bundle pacing for cardiac resynchronization therapy (CRT) and atrioventricular block (AVB) (10). This suggested that HPSP might alleviate tricuspid regurgitation. But its effect on tricuspid regurgitation after left-sided valve surgery remains unclear and awaits further exploration in the future.

Therefore, we retrospectively reviewed patients with tricuspid regurgitation after left-sided valve surgery who underwent HPSP to assess the effect of HPSP on tricuspid regurgitation and heart function.

## Methods

### Study population and data collection

This study was a retrospective study. Patients who underwent permanent cardiac pacemaker implantation of HPSP at Peking University International Hospital from January 1st 2019 to January 1st 2022 were consecutively enrolled. Inclusion criteria: (1) with successful implantation of HPSP; (2) after left-sided heart valve (mitral valve, aortic valve) replacement; (3) with complete baseline and follow-up data.

Clinical data of patients were collected through an electronic medical record system. The baseline data included: (1) Basic information including age, gender, valvular disease history, valve surgery history, AF history, pacing indication, current smoker and drinker; (2) Comorbidity including hypertension, diabetes mellitus, coronary disease, stroke and chronic kidney diseases (CKD); (3) Medications for long-term use which may influence heart function including angiotensin converting enzyme inhibitor (ACEI), angiotensin receptor blocker (ARB), angiotensin receptor-neprilysin inhibitor (ARNI), *β* blockers, spirolactone, loop diuretics and statins; (4) Baseline clinical data at hospitalization before pace maker implantation, including heart function classification of New York Heart Association (NYHA), mean heart rate (HR) from the Holter, systolic blood pressure (SBP), diastolic blood pressure (DBP), hemoglobin, serum creatinine and brain natriuretic peptide (BNP); (5) Electrocardiogram (ECG) characteristics including QRS duration (QRSd); (6) Last preoperative ultrasonic cardiogram (UCG) parameters included left atrial anteroposterior diameter (LAD), left ventricular end-diastolic diameter (LVEDD), left ventricular ejection fraction (LVEF), right atrial left-right diameter (RAD), Right ventricular left-right diameter (RVD), mitral valve regurgitation (MVR) grade, tricuspid valve regurgitation (TVR) grade and velocity, pressure gradient (PG), pulmonary artery systolic pressure (PASP), tricuspid annular plane systolic excursion (TAPSE), right ventricle fractional area change (RV-FAC) and inferior vena cava diameter (IVCD); Estimation of PASP: PASP = 4*(peak velocity of tricuspid regurgitation)^2 ^+ right atrial pressure; (7) Cardiothoracic ratio from last preoperative chest x-ray.

The research was compliance with the Declaration of Helsinki. The study protocol was approved by the Ethics Committee of Peking University International Hospital.

### His-Purkinje system pacing

A C315 delivery sheath (Medtronic, USA) and 3830 electrodes (Medtronic, USA) were used to place the pacing electrodes of the His-Purkinje system. Under the guidance of x-ray, in the right anterior oblique position of 30° (RAO 30°), the tip of the wire was mapped to find the His potential near the tricuspid annulus. When the His potential was mapped, we rotated and fixed the lead, and then measured the pacing parameters. If the His bundle was captured and the threshold was <2.0 V/0.4 ms, the His bundle lead was retained; otherwise, LBBP was performed (11).

Referring to the 9-partition method of the left bundle branch, the LBBP lead was located at or near the basal lower one-third junction of the ventricular fluoroscopic image in the RAO 30° position (12). We threaded the lead proximally to the subintima of the left ventricular septum, and left bundle branch potentials may have been recorded on intracavitary electrograms. The ECG showed a right bundle branch block pattern and usually a W-shape with a notch in lead V1 during pacing. The pacing signal to the peak of the R wave in lead V5 (stimulus to left ventricular activation time, sti-LVAT) shortened abruptly during high output voltage pacing or remained shortest and constant at different output voltage. These can determine the success of LBBP (13, 14).

The right ventricular low-septal pacing electrodes (5076–58, Medtronic, USA) were placed as backup in all patients. The electrodes were connected to the pacemaker (A3DR01, Medtronic, USA). The capture threshold, sensing amplitude, and pacing impedance were recorded. Threshold values were considered with a pulse width of 0.4 ms for all patients. The lower rate for HPSP was set at 60 bpm.

All patients stopped taking medications that lowered their heart rate to determine the pacemaker indication and received adequate and optimized drug therapy before implantation. After implantation, patients with tachycardia took drugs to control the ventricular rate to ensure high pacing ratio. Other long-term medications were also reinstated.

### Follow-up

The patients were followed up by regular clinic visits after implantation, and related data were recorded at the same time. All the patients underwent electrocardiogram, chest x-ray and echocardiography at 3 months after implantation. QRS duration and cardiothoracic ratio were recorded. Echocardiographic parameters same as baseline data were included. Pacing parameters were measured by programming control.

### Statistical analysis

Continuous variables conforming to normal distribution were presented as mean ± SD (standard deviation). Skewed variables were presented as median (interquartile range [IQR]). Discrete variables were presented as frequencies (n) and percentages (%). To compare groups, paired student's t-test was used for normally distributed continuous variables while Mann-Whitney U tests for skewed continuous variables and ordered categorical variables. Linear regression was performed to identify the independent determinants of TVR velocity. All statistical tests were two-tailed. *P* < 0.05 was considered to indicate statistical significance. The SPSS 23 software (IBM, NY, USA) was used.

## Results

### Patient characteristics

**Figure 1:**
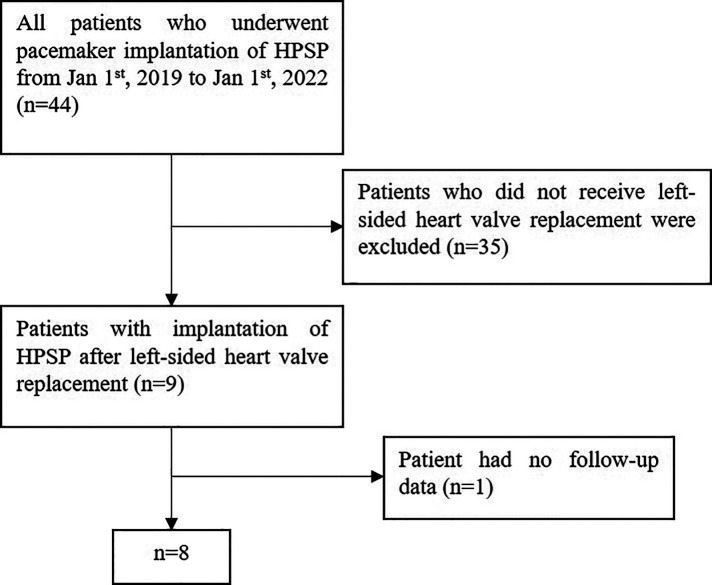
Flowchart of patient inclusion. HPSP, His-Purkinje system pacing; HBP, His bundle pacing; LBBP, left bundle branch pacing.

A total of 44 patients' data was retrospectively reviewed (Figure 1). Patients who did not receive left-sided heart valve replacement were excluded (*n* = 35). One patient who had no follow-up data was excluded. Finally, a total of 8 patients were enrolled in the study, with an average age of 74. Six of the patients were male, and six had rheumatic heart disease. Five patients underwent mitral valve replacement (MVR), two patients underwent aortic valve replacement (AVR), and one patient underwent combined valve replacement. The average postoperative time was 7.8 years. All patients had persistent atrial fibrillation. The average time of onset of AF was 12 years. Three patients underwent pacemaker implantation for symptomatic bradycardia, three for long intermittent and two for complete AVB. Most patients had other chronic diseases such as hypertension, coronary disease, diabetes mellitus, stroke and CKD. All medications which may affect heart function were recorded. The mean heart rate of the patients before implantation was 58.3 ± 12.1 bpm. Baseline characteristics of the patient population are listed in (Table 1). Six patients had class 3 of NYHA and 2 patients had class 2 before implantation (Table 4).

**Table 1:** Patient characteristics.

Variables	*n* = 8
**Basic information**	
Age	74 ± 11
Male (n, %)	6 (75)
Valvular disease (n, %)	Rheumatic 6 (75)
	Senile degenerative 2 (25)
Valve surgery type (n, %)	MVR 5 (62.5)
	AVR 2 (25)
	MVR + AVR 1 (12.5)
Surgery time (years)	7.8 ± 5.0
Persistent AF (n, %)	8 (100)
AF history (years)	12.0 ± 5.1
Pacing indication (n, %)	Symptomatic bradycardia 3 (37.5)
	Long intermittent 3 (37.5)
	Complete AVB 2 (25)
Current Smoker (n, %)	5 (62.5)
Current Drinker (n, %)	5 (62.5)
**Comorbidity**	
Hypertension (n, %)	3 (37.5)
Diabetes mellitus (n, %)	2 (25)
Coronary disease (n, %)	4 (50)
Stroke (n, %)	2 (25)
CKD (n, %)	3 (37.5)
**Medications**	
ACEI/ARB/ARNI (n, %)	4 (50)
β blockers (n, %)	5 (62.5)
Spirolactone (n, %)	2 (25)
Loop diuretics (n, %)	7 (87.5)
Statins (n, %)	6 (75)
**Baseline Clinical Data**	
mean HR (bpm)	58.3 ± 12.1
SBP (mmHg)	141 ± 23
DBP (mmHg)	72 ± 15
Hemoglobin (g/L)	106.6 ± 20.7
Serum creatinine (umol/L)	121.6 ± 48.3
BNP (pg/ml)	602.6 (308.3–1299.1)

Values are mean ± SD, *n* (%), or median (interquartile range).

MVR, mitral valve replacement; AVR, aortic valve replacement; AF, atrial fibrillation; AVB, atrioventricular block; ACEI, angiotensin converting enzyme inhibitor; ARB, angiotensin receptor blocker; ARNI, angiotensin receptor-neprilysin inhibitor; HR, heart rate; SBP, systolic blood pressure; DBP, diastolic blood pressure; BNP, brain natriuretic peptide.

### Pacing parameters during HPSP and follow-up

Of the 8 patients, 3 received HBP, and 5 received LBBP. The QRS duration of post-implantation was longer than that of pre-implantation (101 ± 10 ms vs. 92 ± 10 ms., *P = 0.038*). But all the QRS were still narrow and the duration was less than 120 ms. The QRS duration of LBBP was 92 ± 13 ms at baseline and increased to 102 ± 10 ms after implantation (*P = 0.040*), while the QRS duration of HBP had no change.

We compared the pacing parameters at implantation and at 3-month follow-up. There was no significant difference in sensing amplitude, pacing impedance, and capture threshold between at implantation and at 3-month follow-up among the 8 patients. The pacing threshold of HBP increased during follow-up with no statistical difference (1.7 ± 0.2 V vs. 2.2 ± 0.4 V, *P = 0.083*). The impedance and threshold of LBBP at 3-month follow-up were significantly lower than those at implantation (668 ± 117*Ω* vs.793 ± 98*Ω*, *P *= 0.017; 0.4 ± 0.1 V vs. 0.6 ± 0.1 V, *P = 0.026*), and the sensing amplitude remained stable. The mean heart rate of the patients at 3-month follow-up was 68.1 ± 6.9bpm, which was higher than before implantation (*P = 0.027*). The pacing ratio of the patients was 45.2%-94.0%. Detailed data was listed in the (Table 2).

**Table 2:** QRS duration and pacing parameters.

**Patient No.**	**Pacing type**	**Baseline**	**Pacing**	**At implantation**		
		**QRSd(ms)**	**QRSd(ms)**	**Sensing(mV)**	**Impedance(Ω)**	**Threshold(V)**
1	HBP	94	84	4.2	495	1.5
4	HBP	94	110	3.5	370	1.8
7	HBP	86	98	5.6	570	1.7
*M ± SD*	* *	*91 ± 5*	*97 ± 13*	*4.4 ± 1.1*	*478 ± 101*	*1.7 ± 0.2*
*P value* ^a^	* *		*0.535*			
2	LBBP	80	88	15	890	0.4
3	LBBP	104	106	4.5	890	0.7
5	LBBP	108	114	7.8	790	0.6
6	LBBP	82	104	14.4	675	0.6
8	LBBP	84	100	21	720	0.6
*M ± SD*	* *	*92 ± 13*	*102 ± 10*	*12.5 ± 6.5*	*793 ± 98*	*0.6 ± 0.1*
*P value* ^b^	* *		***0.040***			
*M ± SD*	* *	*92 ± 10*	*101 ± 10*	*9.5 ± 6.5*	*675 ± 187*	*1.0 ± 0.6*
*P value* ^c^	* *	* *	***0.038***	* *	* *	* *
**Patient No.**	**3-month follow-up**					* *
	**Sensing(mV)**	**Impedance(Ω)**	**Threshold(V)**	**Mean HR(bpm)**	**Pacing ratio,%**	
1	5.6	522	1.75	62	82.3	
4	4.5	456	2.6	65	54.3	
7	4.7	550	2.2	66	68.5	
*M ± SD*	*4.9 ± 0.6*	*509 ± 48*	*2.2 ± 0.4*	* *	* *	
*P value* ^a^	*0.554*	*0.418*	*0.083*	* *	* *	
2	12.7	746	0.3	82	45.2	
3	5.8	825	0.5	65	59.1	
5	20	551	0.5	72	48.3	
6	17	570	0.25	61	94.0	
8	16.8	649	0.5	72	61.7	
*M ± SD*	*14.5±5.5*	*668 ± 117*	*0.4 ± 0.1*	* *	* *	
*P value* ^b^	*0.537*	***0.017***	***0.026***	* *	* *	
*M ± SD*	*10.9±6.5*	*609 ± 123*	*1.1 ± 1.0*	*68.1 ± 6.9*	*64.2 ± 16.8*	
*P value* ^c^	*0.450*	*0.105*	*0.549*	***0.027***	* *	

M ± SD: mean ± SD;.

^a^ comparison of HBP.

^b^ comparison of LBBP.

^c^ comparison of all patients.

All comparison was between 3-month follow-up and at implantation or baseline.

*P values* <0.05 in **bold.**

HBP, His bundle pacing; LBBP, Left bundle branch pacing; QRSd, QRS duration.

### Echocardiography, cardiothoracic ratio and NYHA classification

**Figure 2:**
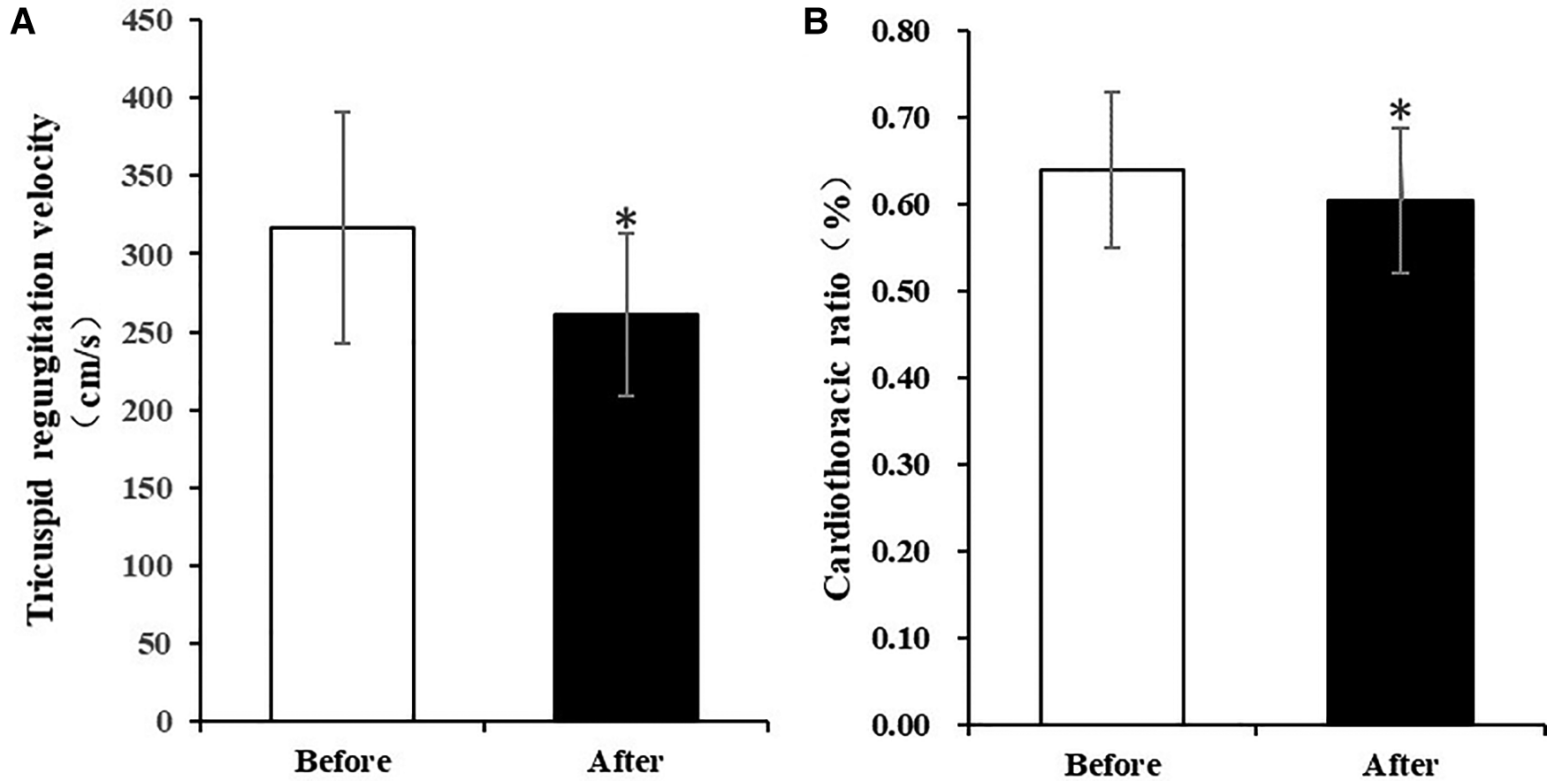
Comparison of tricuspid regurgitation velocity and cardiothoracic ratio before and after implantation of HPSP for 3 months. (**A**) Tricuspid regurgitation velocity significantly decreased after implantation of HPSP for 3 months. (**B**) Cardiothoracic ratio was significantly lower at the 3-month follow-up than that before implantation of HPSP. (*p < 0.05). HPSP, His-purkinje system pacing.

The atrium and ventricle of these patients were enlarged before the pacemaker implantation. The LAD reached 64 ± 18 mm and the RAD reached 48 ± 9 mm. The LVEDD was 53 ± 8 mm and the RVD were 36 ± 7 mm. The LVEF of the patients were approximately normal (62 ± 17%). They suffered from moderate to severe tricuspid regurgitation and had no or mild mitral regurgitation. TAPSE and RV-FAC were in the normal range in these patients. At the 3-month follow-up, the tricuspid regurgitation grade of the patients was significantly lower than that before pacemaker implantation (*P *= 0.007). The tricuspid regurgitation velocity significantly decreased (317 ± 74 cm/s vs. 261 ± 52 cm/s, *P = 0.022*) and tricuspid valve pressure gradient (PG) reduced (42 ± 21mmHg vs. 28 ± 10 mmHg, *P = 0.040*). The estimated PASP was also significantly lower after implantation (52 ± 23 mmHg vs. 37 ± 12 mmHg, *P = *0.028). LAD was reduced to some extent, but the difference was not statistically significant (*P = 0.089*)*.* The diameter of other atria and ventricles, TAPSE, RV-FAC, IVCD did not change significantly compared with that before pacing (Details in Table 3, Figure 2A).

**Table 3:** Changes of echocardiographic parameters.

**Patient No.**	**Before implantation**												
	**LAD**	**LVEDD**	**MVR**	**LVEF**	**RAD**	**RVD**	**TVR**	**V**	**PG**	**PASP**	**TAPSE**	**RV-FAC**	**IVCD**
	**mm**	**mm**		**%**	**mm**	**mm**		**cm/s**	**mmHg**	**mmHg**	**mm**	**%**	**mm**
1	42	44	no	50	63	35	moderate	267	25	38	17	43.2	20
2	55	52	mild	73	41	35	mild	245	29	29	16	55.0	18
3	89	52	no	76	55	34	moderate	341	46	56	18	41.5	20
4	80	64	mild	55	53	44	severe	341	46	61	19	45.3	22
5	42	45	mild	71	35	36	moderate	255	26	36	22	40.2	14
6	58	50	no	69	53	47	severe	476	91	101	21	44.6	24
7	82	65	no	27	46	30	severe	317	40	55	20	38.5	18
8	64	48	moderate	73	40	24	severe	292	34	42	21	52.1	24
*M ± SD*	*64 ± 18*	*53 ± 8*	* *	*62 ± 17*	*48 ± 9*	*36 ± 7*	* *	*317 ± 74 *	*42 ± 21*	*52 ± 23*	*19 ± 2*	*45.1 ± 5.7*	*20 ± 3*
**Patient No.**	**3-month follow-up**												
	**LAD**	**LVEDD**	**MVR**	**LVEF**	**RAD**	**RVD**	**TVR**	**V**	**PG**	**PASP**	**TAPSE**	**RV-FAC**	**IVCD**
	**mm**	**mm**		**%**	**mm**	mm		**cm/s**	**mmHg**	**mmHg**	**mm**	**%**	**mm**
1	45	53	no	52	65	37	mild	229	21	29	21	45.0	18
2	38	46	mild	63	43	33	mild	224	20	28	17	61.3	18
3	71	49	no	56	53	35	mild	300	36	46	16	37.5	22
4	82	74	mild	58	67	42	moderate	323	42	52	20	40.1	20
5	42	47	no	73	43	36	mild	251	25	30	18	44.2	16
6	55	53	no	60	47	47	moderate	331	44	54	22	43.6	21
7	77	63	no	43	48	33	mild	183	14	22	19	40.5	14
8	57	43	mild	60	42	26	mild	249	25	33	20	50.7	25
*M ± SD*	*58 ± 17*	*54 ± 10*	* *	*58 ± 9*	*51 ± 10*	*36 ± 6*	* *	*261 ± 52*	*28 ± 10*	*37 ± 12*	*19 ± 2*	*45.4 ± 7.6*	*19 ± 3*
*P value*	*0.089*	*0.657*	*0.511*	*0.400*	*0.239*	*0.470*	***0.007***	***0.022***	***0.040***	***0.028***	*0.888*	*0.829*	*0.390*

M ± SD: mean ± SD; *P values* <0.05 in **bold.**

LAD, left atrial anteroposterior diameter; LVEDD, left ventricular end-diastolic diameter; MVR, mitral valve regurgitation; LVEF, left ventricular ejection fraction; RAD, right atrial left-right diameter; RVD, right ventricular left-right diameter; TVR, tricuspid valve Regurgitation; V, tricuspid regurgitation velocity; PG, pressure gradient; PASP, pulmonary artery systolic pressure; TAPSE, tricuspid annular plane systolic excursion; RV-FAC, right ventricle fractional area change; IVCD, inferior vena cava diameter.

Three months after pacemaker implantation, the cardiothoracic ratio of patients was significantly lower than that before implantation (0.61 ± 0.08 vs. 0.64 ± 0.09, *P = 0.017*) (Table 4, Figure 2B). The activity tolerance of most patients improved after pacing, and the NYHA classification also improved (*P = 0.013*). Six patients had class 2 of NYHA, one had class 1 and one had class 3 (Table 5).

**Table 4:** Changes of cardiothoracic ratio and NYHA classification.

Patient No.	Cardiothoracic ratio		NYHA	
	Baseline	3-months follow-up	Baseline	3-months follow-up
1	0.59	0.59	2	2
2	0.58	0.57	3	2
3	0.64	0.64	2	1
4	0.75	0.70	3	3
5	0.47	0.43	3	2
6	0.71	0.65	3	2
7	0.70	0.67	3	2
8	0.68	0.59	3	2
*M ± SD*	0.64 ± 0.09	0.61 ± 0.08		
*P value*	* *	***0.017***	* *	***0.013***

M ± SD: mean ± SD; *P values* <0.05 in **bold.**

**Table 5:** Univariate linear regression analysis for variation of tricuspid regurgitation velocity.

Factors	β	*P value*
**Basic information**		
Age	−0.367	0.371
Gender	0.369	0.369
Valvular disease	0.495	0.212
Type of valve surgery	0.495	0.212
Surgery time (years)	−0.080	0.850
AF history (years)	−0.195	0.644
Current Smoker	−0.152	0.719
Current Drinker	−0.152	0.719
**Comorbidities**		
Hypertension	−0.404	0.320
Diabetes mellitus	−0.369	0.369
coronary disease	−0.100	0.814
Stroke	−0.288	0.489
CKD	0.018	0.966
**Medications**		
ACEI/ARB/ARNI	0.070	0.869
β blockers	−0.090	0.832
Spirolactone	0.369	0.369
Loop diuretics	0.260	0.534
Statins	−0.415	0.307
**Baseline Clinical Data**		
SBP	0.211	0.617
DBP	−0.303	0.465
Hemoglobin	0.002	0.996
Serum creatinine	−0.133	0.754
BNP	−0.260	0.534
**Pacing parameters**		
Pacing type	−0.121	0.775
mean HR variation	0.105	0.804
QRSd variation	−0.447	0.267
Pacing ratio	0.736	**0.037**
**UCG parameters**		
LAD variation	−0.048	0.909
LVEDD variation	0.092	0.828
LVEF variation	−0.232	0.580
RAD variation	0.625	**0.097**
RVD variation	−0.439	0.276
TAPSE variation	−0.129	0.762
RV-FAC variation	0.079	0.852
IVCD variation	0.711	**0.048**

β: standardized regression coefficient. *P* values <0.2 in **bold**.

variation = the baseline value - the follow-up value.

AF, atrial fibrillation; ACEI, angiotensin converting enzyme inhibitor; ARB, angiotensin receptor blocker; ARNI, angiotensin receptor-neprilysin inhibitor; SBP, systolic blood pressure; DBP, diastolic blood pressure; HR, heart rate;.

QRSd, QRS duration; UCG, ultrasonic cardiogram; LAD, left atrial anteroposterior diameter; LVEDD, left ventricular end-diastolic diameter; LVEF, left ventricular ejection fraction; RAD, right atrial left-right diameter; RVD, right ventricular left-right diameter; TAPSE, tricuspid annular plane systolic excursion; RV-FAC, right ventricle fractional area change; IVCD, inferior vena cava diameter.

### Multivariate liner regression analysis

There were many factors affecting tricuspid regurgitation. A univariate regression for the variation of tricuspid regurgitation velocity variation was performed to identify the influencing factors (Table 5). The variation was equal to the baseline value minus the follow-up value. Basic information, comorbidities, medications, baseline clinical data, pacing parameters and UCG variation were all included for analysis. Factors with *P* < 0.2 in the univariate regression analysis (pacing ratio, RAD variation and IVCD variation) and factors commonly considered to be associated with tricuspid regurgitation and heart failure (valve surgery type, surgery time, AF history, anti-heart failure medications, pacing type, mean HR variation, QRSd variation, LVEF variation, TAPSE variation and RV-FAC variation) were included in the multivariate linear stepwise regression analysis (Table 6). We found that pacing ratio (*β *= 0.736, *P* = 0.037) was the only independent determinant of tricuspid regurgitation velocity variation.

**Table 6:** Multivariate linear regression analysis for variation of tricuspid regurgitation velocity.

Factors	β	*P value*
**Basic information**		
Valve surgery type	0.042	0.920
Surgery time (years)	−0.240	0.446
AF history (years)	−0.183	0.557
**Medications**		
β blockers	−0.078	0.805
Loop diuretics	−0.095	0.789
ACEI/ARB/ARNI	0.233	0.460
Spirolactone	0.379	0.192
**Pacing parameters**		
Pacing type	0.032	0.921
mean HR variation	0.138	0.662
QRSd variation	−0.403	0.159
Pacing ratio	0.736	0.037
**UCG parameters**		
LVEF variation	−0.179	0.568
TAPSE variation	0.335	0.342
RV-FAC variation	−0.078	0.811
RAD variation	0.283	0.468
IVCD variation	0.415	0.272

β: standardized regression coefficient. 95% CI: 95% confidence interval; *P* values <0.05 in **bold**.

AF, atrial fibrillation; ACEI, angiotensin converting enzyme inhibitor; ARB, angiotensin receptor blocker; ARNI, angiotensin receptor-neprilysin inhibitor; HR, heart rate; QRSd, QRS duration; UCG, ultrasonic cardiogram; LVEF, left ventricular ejection fraction; RAD, right atrial left-right diameter; TAPSE, tricuspid annular plane systolic excursion; RV-FAC, right ventricle fractional area change; IVCD, inferior vena cava diameter.

### Typical case

**Figure 3:**
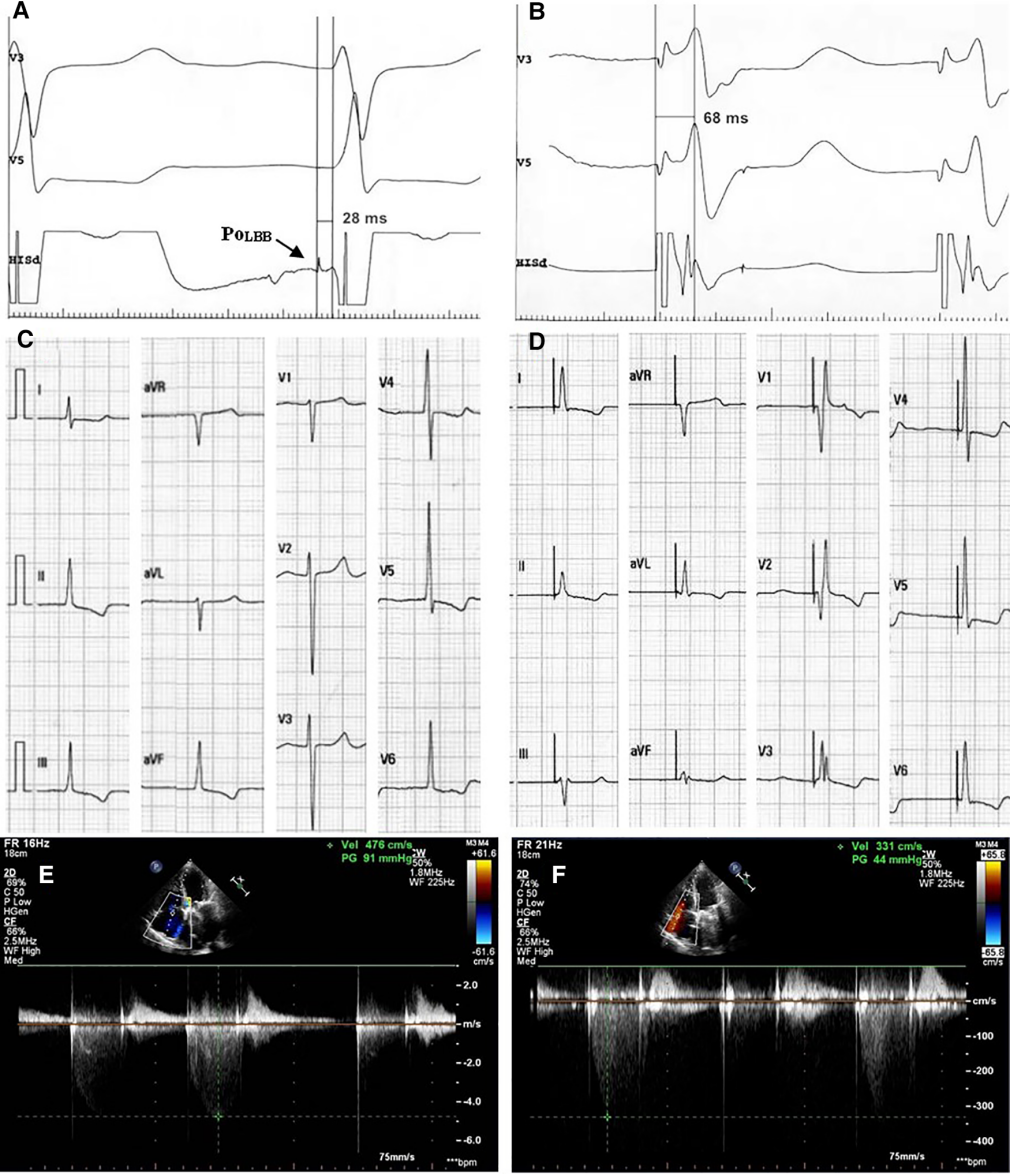
Clinical data of typical case. (**A**) Left bundle branch potential; (**B**) LBBP: sti-LVAT was 68 ms; (**C**) ECG before implantation; (**D**) ECG after implantation; (**E**) Echocardiography before implantation: tricuspid regurgitation velocity 476 cm/s; (**F**) Echocardiography at 3-month follow-up: tricuspid regurgitation velocity 331 cm/s. PoLBB, potential of left bundle branch; LBBP, left bundle branch pacing; sti-LVAT, stimulus to left ventricular activation time; ECG, electrocardiogram.

Case 6 was a 65-year-old male, who was admitted to the hospital because of rheumatic valvular heart disease and heart failure. His chief complaint was shortness of breath after exercise and edema of lower extremity for more than 30 years, which was aggravating for one month. The patient underwent mechanical mitral valve replacement 6 years ago. After adequate diuretic treatment, the patient's symptoms were not completely resolved. The echocardiography showed that the mechanical mitral valve worked well and LVEF was 69%. But he had severe tricuspid regurgitation and his regurgitation velocity was 476 cm/s (Figure 3E). The electrocardiogram showed atrial fibrillation and heart rate was 40–50 bpm. The patient had definite bradycardia and was advised to implant a permanent pacemaker. During the implantation, the left bundle branch potential was captured (Figure 3A) and LBBP was successfully performed. The sti-LVAT was 68 ms (Figure 3B) and paced QRS duration was 104 ms (Figure 3D). After 3 months of follow-up, the patient's symptoms of heart failure were significantly improved. Echocardiography showed that tricuspid valve regurgitation reduced to moderate and the regurgitation velocity decreased to 331 cm/s (Figure 3F). The pacemaker programming found that the capture threshold was lower than that at implantation and the pacing ratio was 94%.

## Discussion

In our study, we found that HPSP reduced tricuspid regurgitation grade, velocity and PG in patients with persistent atrial fibrillation after left-sided valve replacement and improved heart function. The pacing ratio of HPSP was independently associated with tricuspid regurgitation velocity, which indicated heart rate control and rhythm regularization of atrial fibrillation may be principal reason for the effect.

The mechanism of tricuspid regurgitation after left heart valve surgery was unclear. Severe left heart valve disease caused elevated left atrial pressure and pulmonary hypertension, leading to right heart insufficiency, tricuspid annular dilatation, and subsequent tricuspid regurgitation. However, the above-mentioned mechanism could not explain occurrence of tricuspid regurgitation after left heart valve surgery, because severe pulmonary hypertension and heart failure had significantly improved in most patients after successful valve surgery. Several years later, these patients gradually developed severe tricuspid regurgitation, while the left heart valve function remained normal, and there was no obvious left heart failure (2–5). As in our study, the patients' left ventricular function was basically normal but they had moderate to severe tricuspid regurgitation. Therefore, tricuspid regurgitation may not relate to valve surgery and rheumatic disease in our study. Some retrospective studies found that atrial fibrillation was considered to be main cause and an independent risk factor for the occurrence and progression of tricuspid regurgitation for the patients after left-sided valve surgery (3, 15, 16). Animal and clinical studies had shown that atrial fibrillation with tachycardia, bradycardia or irregular rhythm could lead to cardiac insufficiency (17–19). Chronic atrial fibrillation promoted atrial remodeling and further dilation of the tricuspid annulus, leading to the development of tricuspid regurgitation (3). Moreover, the MAZE operation could prevent the progression of tricuspid regurgitation by reducing AF in these patients with previous mitral or combined mitral/aortic valve surgery (20, 21). Thus, AF may be an important reason for tricuspid regurgitation after left-sided valve surgery.

The treatment of valvular atrial fibrillation was difficult. Because of the fibrosis of the atrial matrix, radiofrequency ablation was ineffective and it was difficult to maintain sinus rhythm (22). Several studies suggested that HPSP could be used in the treatment of atrial fibrillation combined with heart failure. Huang et al. (23) found permanent HBP combined with atrioventricular node (AVN) ablation significantly improved echocardiographic LVEF and NYHA classification and reduced diuretics use in atrial fibrillation patients with heart failure. Wang et al. (24) analyzed patients with persistent AF and heart failure who had indications for implantable cardioverter-defibrillator (ICD) implantation and found that HPSP combined with AVN ablation can improve LV function and reduce the incidence of inappropriate shocks. These findings of improved clinical outcomes are similar to that of HBP and LBBP in heart failure patients with or without AF (9, 25). HPSP combined with AVN ablation not only provided heart rate control and rhythm regularization but also maintained the ventricular electrical and mechanical synchronization, which could finally improve left heart function. In our study, eight patients suffered from persistent atrial fibrillation combined with tricuspid regurgitation. They were treated with HPSP for symptomatic bradycardia, long intermittent and complete AVB. The patients didn't receive AVN ablation because the mean ventricular rate was low. The mean heart rate increased after HPSP. Those who had tachycardia took drugs to control the rate, so the overall pacing ratio of HPSP was more than 40% (45.2%-94.0%). Our study found that HPSP could significantly reduce tricuspid regurgitation. And the pacing ratio was an independent determinant of tricuspid regurgitation velocity. Therefore, we speculated HPSP may improve tricuspid regurgitation for the similar reason as that of patients without valve surgery. HPSP combined with medication achieved rate control and rhythm regularization which may increase cardiac output and improve left heart function, thereby reducing tricuspid regurgitation. The effect could not be simply explained by increasing heart rate, as mean heart rate variation was not an independent factor for tricuspid regurgitation velocity in multiple regression analysis. Besides, in our study, the QRSd of all patients before and after implantation was less than 120 ms. This indicated that HPSP maintained the ventricular synchronization, which may not improve but at least maintain heart function.

The traditional understanding was that the electrodes across the tricuspid valve mechanically prevented the valve from closing, which was the primary reason for tricuspid regurgitation of right ventricular pacing (RVP) (26). However, tricuspid regurgitation was able to improve when the electrodes implanted at the right ventricular septum compared with that at the right ventricular apical (27). Studies had shown that complications such as tricuspid regurgitation and new-onset atrial fibrillation occurred only when the ventricular pacing ratio exceeded 40% (28, 29). These all suggested that ventricular electrical and mechanical dyssynchrony may be the key cause for TR, whether the electrodes cross the valve or not. Recently, Grieco et al. (30) explored the influence of HBP and RVP on right heart function and found that compared with RVP, although some HBP electrodes crossed the tricuspid valve and were located under the valve, parameters of right heart function such as tricuspid regurgitation, PASP, TAPSE and RV-FAC were significantly improved at 6 months. A study using 3D echocardiography showed moderate-to-severe tricuspid regurgitation reduced to mild in patients after HBP, even though the electrodes crossed the tricuspid valve (31). Moreover, the most obvious difference between LBBP and HBP was that the electrode of LBBP was 100% implanted across the tricuspid valve into the ventricular septum, while the electrode of HBP could be implanted at the right atrium side without across the tricuspid valve. Therefore, it had been suggested that LBBP was more likely to induce or worsen tricuspid regurgitation than HBP (32, 33). But the current findings did not support the hypothesis. A large sample size of single-center data showed 618 patients underwent LBBP followed up for 18.6 months and found that 31.4% of the patients had improved tricuspid regurgitation grade, and the number of patients with moderate to severe tricuspid regurgitation decreased at one year (34). Just like the findings in our study, HBP and LBBP did not worsen, but they helped to reduce tricuspid regurgitation. We speculated that HPSP improved left ventricular function and canceled out part of the influence that the electrodes had on the tricuspid valve.

There were some differences in pacing function between HBP and LBBP. HBP could achieve a physiological pacing and the paced ECG was close to normal. But there were problems such as difficult implantation, low sensing amplitude and high pacing threshold (35). The left bundle branch had a relatively large range for implantation. Huang et al. first demonstrated the direct capture of left bundle by placing the lead deep inside the septum resulting in synchronized activation of ventricles, which had low and stable capture thresholds over follow-up (36). Previous studies suggest that the clinical role of LBBP was similar to HBP (25, 37). In our study, patients accepted HBP group had a shorter QRS duration but a higher threshold, and the threshold increased during follow-up. Meanwhile, the threshold and impedance in the LBBP group were decreased during follow-up. Therefore, compared with HBP, LBBP may be a very promising alternative to HBP as a method for delivering physiological pacing.

### Limitations

Firstly, the sample size of our study was relatively small, because there were rare patients with after left-side valve replacement combined with pacing indication. We consecutively enrolled all patients who received HPSP in our heart center in the last 3 years. The samples were assumed to be representative. And at 3 months follow-up, the tricuspid regurgitation grade, flow velocity and PG were all significantly reduced compared with those before pacing, which may be not by chance. Secondly, HPSP was not compared with traditional RVP in our study. Due to the popularity of physiological pacing in recent years, RVP was not routinely recommended for patients with atrial fibrillation complicated with heart failure or tricuspid regurgitation. From the results of the current studies, HPSP may be the better choice. Thirdly, we did not find the direct effect of HPSP on left and right heart function perhaps due to the short follow-up time. Future study could include a larger sample size and longer follow-up to explore the effect of HPSP on tricuspid regurgitation and heart function in patients with persistent atrial fibrillation after left-sided valve surgery.

## Conclusion

Our study found that HPSP might reduce tricuspid regurgitation and improve heart function for patients with persistent atrial fibrillation after left-sided valve surgery, which may be associated with heart rate control and rhythm regularization of atrial fibrillation. This may be a novel treatment option for patients with tricuspid regurgitation after left-sided valve surgery who were at high risk of reoperation.

## Data Availability

The raw data supporting the conclusions of this article will be made available by the authors, without undue reservation.
